# A Combination of Human Embryonic Stem Cell-Derived Pancreatic Endoderm Transplant with LDHA-Repressing miRNA Can Attenuate High-Fat Diet Induced Type II Diabetes in Mice

**DOI:** 10.1155/2015/796912

**Published:** 2015-12-03

**Authors:** Yunya Chen, Xiujie Wang, Xinyu Shao

**Affiliations:** ^1^Department of Endocrinology, Wuxi People's Hospital of Huishan District, 2 Zhanqian Street, Wuxi, Jiangsu 214187, China; ^2^Department of Endocrinology, The First Affiliated Hospital of Soochow University, 188 Shizi Street, Suzhou, Jiangsu 215006, China

## Abstract

Type II diabetes mellitus (T2D) is a chronic metabolic disorder that results from defects in both insulin secretion and insulin action. The deficit and dysfunction of insulin secreting *β*-cell are signature symptom for T2D. Additionally, in pancreatic *β*-cell, a small group of genes which are abundantly expressed in most other tissues are highly selectively repressed. Lactate dehydrogenase A (LDHA) is one of such genes. Upregulation of LDHA is found in both human T2D and rodent T2D models. In this study, we identified a LDHA-suppressing microRNA (hsa-miR-590-3p) and used it together with human embryonic stem cell (hESC) derived pancreatic endoderm (PE) transplantation into a high-fat diet induced T2D mouse model. The procedure significantly improved glucose metabolism and other symptoms of T2D. Our findings support the potential T2D treatment using the combination of microRNA and hESC-differentiated PE cells.

## 1. Introduction

People with obesity (body mass index above 25–29.9 kg/m^2^) are more prone to morbidity of hypertension, coronary heart disease, and type II diabetes (T2D) [1]. In addition to genetic disposition, overnutrition has also been suspected as a critical factor to induce obesity and T2D [1–3]. It has been reported that high-fat diet led to not only obesity via the expanding of fat storage, but also hyperglycemia which gradually resulted in notable insulin resistance [4, 5]. The patients suffering from T2D were reported to exhibit progressively decreased number of pancreatic insulin-positive *β*-cells as well as their function [6]. Mature *β*-cells have the exclusive task to produce insulin [7]. Numerous genes that are required for glucose-induced insulin secretion and cells survival are highly or selectively expressed in *β*-cells [8]. In addition, proper control of glucose-induced insulin secretion also involves the absence or the low expression of  “disallowed genes” including those coding for lactate dehydrogenase A (LDHA) and monocarboxylate transporter-1 (MCT1) [9]. Overexpression of LDHA in insulin secreting cells affects glucose-induced insulin secretion. Islets of individuals with diabetes display an increase in the expression of LDHA when compared to controls [10–12].

Advances in stem cell technology have provided exciting development in diabetes treatments. For example, stem cells, such as cord blood stem cells, exhibited immunomodulatory capacity in the treatment of type I diabetes [13, 14]. Particularly interesting to our current study, human embryonic stem cells (hESCs) were used to differentiate to pancreatic endoderm (PE) capable of treating diabetic mice [15, 16]. Pagliuca et al. have even demonstrated in a very recent study the large scale* in vitro* differentiation of human embryonic stem cells (hESCs) to generate glucose-responsive insulin-producing pancreatic *β*-cells [17].

In this study, we first identified microRNA hsa-590-3p which specifically targets LDHA. Then, we transduced this microRNA into H1 ESC line using a tet-on system. Without doxycycline induction, microRNA transduced H1 ESCs were differentiated to PE cells (miR-PE). miR-PE cells were transplanted into mice and the effect on their diabetic symptoms was assessed. We aimed to provide a proof of concept of using a combination of stem cell and microRNA therapy to treat T2D.

## 2. Materials and Methods

### 2.1. Animals and Transplant of hESC-Derived Cells

Eighty 4-week-old male C57BL/6J mice were employed for this study. The care and use of animals in this study followed the guidelines and protocol approved by the Institutional Animal Care and Use Committee (IACUC) of The First Affiliated Hospital of Soochow University. The IACUC committee members at The First Affiliated Hospital of Soochow University approved this study. All efforts were made to minimize the number of animals used and their suffering. They were kept in a temperature (21 ± 2°C) and humidity (55 ± 10%) controlled room on a 12 h : 12 h light : dark cycle (light 7 AM–7 PM).

The animals were subdivided into four groups (20 mice in each group): sham transplant (the same surgical procedure but no actual transplant) with high-fat diet (Sham + HFD) as positive control, sham transplant with normal diet (Sham + ND) as baseline control, normal PE transplant with high-fat diet (PE + HFD), and hsa-miR-590-3p transfected PE transplant with high-fat diet (miR-PE + HFD).

The animals were anesthetized with inhalable isoflurane and received transplants of 5 × 10^6^ stage 4 hESC-derived PE cells (with or without hsa-miR-590-3p transduction) under the left kidney capsule 1 week after they arrived at the animal holding facility. All mice were administered with oral enrofloxacin (100 mg/mL in drinking water) (Bayer Animal Health) for 1 week after transplant.

The animals had* ad libitum* access to water and a standard normal diet (ND) or high-fat diet (HFD) (Research Diets, New Brunswick, NJ, USA) for 16 weeks after PE transplantation. On caloric basis, the high-fat diet consisted of 58% fat from lard, 25.6% carbohydrate, and 16.4% protein (total 23.4 kJ/g), whereas the normal diet contained 11.4% fat, 62.8% carbohydrate, and 25.8% protein (total 12.6 kJ/g).

Doxycycline hydrochloride (Sigma-Aldrich, Steinheim, Germany) was prepared as a 50 mg/mL stock solution in 50% ethanol and supplied with the drinking water at a final concentration of 1 mg/mL at the start of the experiment. The doxycycline-containing water was renewed every 2-3 days.

### 2.2. Cultivation and Differentiation of H1 ESC

Human embryonic stem cells (hESCs) H1 were used in this study. H1 ESC is NIH registered and provided by WiCell Research Institute. H1 ESCs were cultured using mTeSR 1 feeder-free system on Matrigel (Becton, Dickinson and Company, Franklin Lakes, NJ, http://www.bd.com/) coated plate following the manufacturer's instructions. The H1 ESCs were maintained and passaged as previously described [18]. The four-stage differentiation protocol was carried out as previously described [15]. In stage 1, H1 hESCs were differentiated towards definitive endoderm (DE). H1 hESCs were washed briefly with 1x phosphate buffered saline (PBS) at 75% confluency and then cultured with RPMI medium containing activin (100 ng/mL) supplemented with 0, 0.2%, and 0.2% (v/v) FBS (Biowest) on days 1–3, respectively. Wnt3a (25 ng/mL) was added to day 1 medium to improve the transition. In stage 2, DE was further transformed to primitive gut (PG) tube. DE was cultured for 3 days with RPMI medium containing 2% FBS and supplemented with FGF10 (50 ng/mL) and KAAD-cyclopamine (CYC) (20 ng/mL). In stage 3, PG cells were treated with retinoic acid (RA) (0.5 *μ*M) together with CYC (20 ng/mL) and FGF10 (50 ng/mL) in DMEM supplemented with B27 (Invitrogen) (1%) for 3 days to become PDX1-expressing posterior foregut (PF) endoderm cells. In stage 4, PF cells were further transitioned into pancreatic and endocrine lineages. During 3 days of culture, RA was removed from the medium and DAPT (1 *μ*M) and exendin-4 (Ex4) (50 ng/mL) were added to the medium. Cells after stage 4 were characterized by coexpression of PDX1, FOXA2, and SOX9 and were referred to as pancreatic endoderm (PE).

### 2.3. Immunofluorescence

Cells were washed with 1x PBS for three times and fixed with 4% paraformaldehyde (PFA) for 10 minutes at room temperature, followed by 10-minute permeabilization with 0.2% Triton X-100 in 1x PBS. The cells were blocked for 1 hour with 5% goat serum and 2% bovine serum albumin. Diluted primary antibodies were then added to the culture and incubated at 4°C overnight. IgG isotype control mouse and rabbit polyclonal IgG (Abcam, UK) were used as control for staining, respectively. After 4 washes with 1x PBS, diluted secondary antibodies against specific primary antibodies were added and incubated in dark at room temperature for 1 hour. 4′,6-Diamidino-2-phenylindole (DAPI, Invitrogen) was added 10 minutes before the final wash. Pictures were taken under fluorescent microscope. The primary antibodies used were OCT4 (mouse, 1 : 200 dilution, Life Technologies), SOX9 (rabbit, 1 : 300 dilution, Life Technologies), PDX1 (rabbit, 1 : 300 dilution, Life Technologies), and FOXA2 (mouse, 1 : 200 dilution, Life Technologies). The secondary antibodies used were Alex 488 (goat anti-mouse, 1 : 500, Life Technologies) and Alexa 568 (goat anti-rabbit, 1 : 500, Life Technologies).

### 2.4. Immunohistochemistry of the Pancreas

Mice were sacrificed by cervical dislocation. Pancreas was rapidly removed and fixed with 10% formalin and embedded in paraffin. The paraffin sections were dewaxed, rehydrated, and digested with 0.05% trypsinase at 37°C for 15 min. After being blocked by 10% goat serum in 1x phosphate buffered saline (1x PBS) for 3 h at room temperature (RT), all sections were incubated overnight at 4°C with polyclonal guinea pig anti-swine insulin (1 : 100; DakoCytomation, Carpinteria, CA). In the second day, the sections were first conducted in the blocking solution at RT, followed by washing twice in PBS before reacting with 1 : 200 Alexa Fluor H 488 conjugated goat anti-guinea pig IgG antibodies (Molecular Probes, Eugene, OR) in the blocking solution for 2 h at 37°C. Following two washes with PBS, DAPI (4,6-diamidino-2-phenylindole, 0.5 mg/mL in PBS) was added to the slides and the slides were washed again after 5 min. All slides were then mounted with SlowFade Antifade Medium (Invitrogen, Carlsbad, CA), examined, and recorded blindly by two investigators using an epifluorescence microscope (6100; Olympus, Tokyo, Japan) with a grey level CCD (charge coupled device) camera (Olympus).

*β*-cell mass was estimated as the product of the total cross-sectional *β*-cell area over total tissue area and the weight of the pancreas before fixation. Islet area was traced manually and processed using Image-Pro Plus 6.0 software (Media Cybernetics, Silver Spring, USA) to measure islet size distribution.

### 2.5. MicroRNA Transfection

SMARTchoice human inducible lentiviral hsa-miR-590-3p shMIMIC was purchased from GE Lifesciences (Product ID: VSH6904-224644358) along with SMARTchoice human inducible nontargeting control (Product ID: VSC6847). Both microRNA and negative control were transduced into cell lines according to the manufacturer's instructions.

### 2.6. Luciferase Reporter Assay

The putative hsa-miR-590-3p binding site at the 3′-UTR of LDHA was cloned downstream of SV40 promoter-driven Gaussia luciferase (GLuc) reporter gene in pEZX-MT05 vector (GeneCopoeia). Mutant forms of the luciferase constructs were also generated using standard PCR-based overlap-extension protocols. For luciferase reporter assay, HeLa cells (3 × 10^4^) were plated in a 24-well plate and then cotransfected with 400 ng of either hsa-miR-590-3p or miR-control (Applied Biosystems), 200 ng of either wild-type luciferase construct, using Lipofectamine 2000 (Invitrogen) according to the manufacturer's instruction. Cells were collected 48 h after transfection and analyzed using the Dual-Luciferase Reporter Assay System (Promega). The pEZX-MT05 vector also contains a secreted Alkaline Phosphatase (SeAP) reporter driven by a CMV promoter and served as the internal control to correct the differences in transfection and harvest efficiencies. Data are shown as mean ± SEM of three independent experiments.

### 2.7. Measurements of Serum Glucose and Insulin Levels

Mice were anesthetized using isoflurane and euthanized and blood was collected from arteriovenous vessels. The blood samples were then centrifuged by microcentrifuge method to gain sera that were then stored in freezer at −70°C until the evaluation of glucose and insulin levels. The concentrations of total cholesterol (TC) and triglyceride (TG) were determined using a commercial kit (Asan Pharmaceutical Co., Seoul, Korea) based on the enzymatic colorimetric method.

### 2.8. Body Weight, Composition, and Metabolic Assessment

Body weight, blood glucose, and plasma insulin were measured prior to PE transplant (week 0) and every 4 weeks up to week 16. Body weight was measured on a top-loading balance (Accu-622; Fisher Scientific), and nonfasting blood samples were obtained via tail venipuncture to determine insulin and glucose levels. Blood glucose levels were determined by glucometer (Lifescan Surestep) and plasma insulin levels were quantified by ELISA.

At the end of the study, body composition (lean mass and fat content) was measured using a dual-energy X-ray absorptiometry method (DEXA, Sabre Bone Densitometry; Norland Med, WI). Plasma insulin and leptin were assayed using rat insulin ELISA kits (Crystal Chem Inc., IL) and mouse leptin ELISA kits (Crystal Chem Inc., IL), respectively. Plasma free fatty acids (FFAs) and triglycerides (TGs) were assessed using Wako NEFAC (Wako Chemicals USA, Inc., Richmond, VA) and Free Glycerol Reagent (Sigma, MO).

### 2.9. Liver Weight and Liver TG Content

Mice were sacrificed to dissect and weigh liver tissue at the end of 16-week experiment. Liver TG content was measured by homogenizing liver in chloroform/methanol (vol : vol 2 : 1) and incubated at room temperature for 4 hours. The tissue lysate was then dried in air and resuspended by KOH (3 M) for 1 hour incubating at 70°C. MgCl_2_ was used to neutralize the lysate and TG assay was performed as described above.

### 2.10. Real-Time Quantitative Reverse Transcriptase-Polymerase Chain Reaction (RT-PCR) Analysis

Total RNA from isolated pancreas were extracted by RNeasy Mini Kit (Qiagen). Quality and quantity of RNA were measured by NanoDrop 8000 spectrophotometer (Thermo Scientific). 1,000 ng RNA was used for each reaction to produce cDNA using high capacity cDNA reverse transcription kit (Applied Biosystems) following the manufacturer's instructions. cDNA products were diluted by adding RNase- and DNase-free water and frozen at −20°C before gene expression assay. Each PCR reaction mixture contained 10 *μ*L 1x PCR master mix, 5 *μ*L diluted cDNA, 4 *μ*L water, and 1 *μ*L probe. Gene expression was measured with quantitative real-time RT-PCR system.

### 2.11. Western Blotting

The expressions of insulin, GLUT2, PGC-1*α*, GCK, and G6Pase were determined by western blot and GAPDH was employed as control. Pancreas was lysed using cell lysis reagent (Sigma) and phosphatase inhibitors (Sigma) and lysed by Tissue Homogenizer (Bertin Technologies, France). The crude lysate was transferred to new Eppendorf tubes. Each sample was added to 20 *μ*L 2x sample loading buffer (0.125 M of 5 M Tris-HCl, Amresco; 20% glycerol, Usb; 4% of 10% sodium dodecyl sulfate, Amresco; 1%  *β*-mercaptoethanol, Amresco; 0.2% of 0.05% (w/v) bromophenol blue, Sigma) and boiled for 5 min before loading. Proteins were separated by SDS-PAGE, transferred to immobilon P membrane (Millipore), and probed with different antibodies as indicated. All antibodies were purchased from Cell Signaling (Beverly, MA). The results were visualized using ECL kit (Abcam) and observed by GeneGnome machine (Syngene).

### 2.12. Statistical Analysis

All data were analyzed using Prism version 5 software (GraphPad, San Diego, CA). Data are presented as mean ± SD. Comparisons of two groups were made by unpaired Student's *t*-test and one-way ANOVA and *p* < 0.05 was considered statistically significant.

## 3. Results

### 3.1. miR-590-3p Directly Targets and Downregulates LDHA Levels in HeLa Cell Line

**Figure 1:**
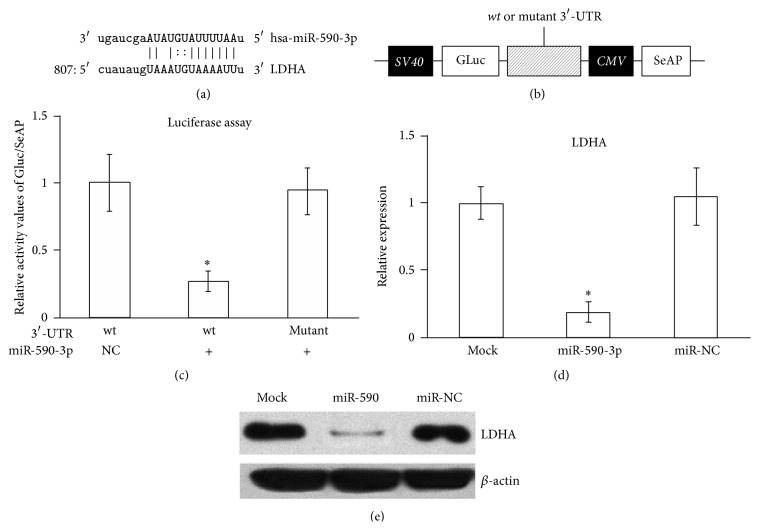
Sequence alignment of hsa-miR-590-3p and* LDHA* mRNA and confirmation of inhibitory function of hsa-miR-590-3p on LDHA using luciferase assay and HeLa cells. (a) Sequence alignment of hsa-miR-590-3p and LDHA mRNA. (b) The construct for luciferase assay. (c) The relative activity of GLuc/SeAP in luciferase assay. (d) Real-time qPCR results of LDHA expression in HeLa cells transfected with no miR, miR-590, and miR-NC. (e) Western blot results for LHDA protein in HeLa cells transfected with no miR, miR-590, and miR-NC. All values are given as mean; error bar represents standard error. ^*∗*^ *p* < 0.05.

Using microRNA.org as resource, we identified miR-590-3p as a potential regulator of* LDHA* mRNA. As shown in Figure 1(a), miR-590-3p recognized 1 locus in the 3′-UTR of* LDHA* (NM_005566) at 807 (Figure 1(a)).

To confirm that LDHA is a bona fide target of hsa-miR-590-3p, luciferase reporter assay was performed, using sequences from original 3′-UTR on* LDHA* mRNA as well as mutated versions (MUT) (Figure 1(b)). MUT contained mutations that disrupted only the potential binding site on LDHA 3′-UTR. As a result, luciferase activity was dramatically reduced by cotransfection of hsa-miR-590-3p, to less than 30% of miR-control transfected levels (Figure 1(c)), indicating that* LDHA* 3′-UTR was indeed a direct target of hsa-miR-590-3p.

We next transfected hsa-miR-590-3p into HeLa cell lines and analyzed both mRNA and proteins levels of LDHA. Compared with miR-control transfections, introducing hsa-miR-590-3p significantly reduced mRNA levels of* LDHA* (Figure 1(d)), suggesting that regulation of LDHA by hsa-miR-590-3p occurred mainly through mRNA degradation rather than translation repression. Then, using antibody against LDHA, we were able to detect that its protein levels were consistently downregulated in hsa-miR-590-3p transfected HeLa cell lines, but not in miR-control transfected experiments (Figure 1(e)). Taken together, the above results clearly demonstrated that hsa-miR-590-3p downregulated* LDHA* mRNA* in vitro* in HeLa cell lines.

### 3.2. miR-590-3p Suppresses LDHA Expression in hESC-Derived PE Cells

H1 ESC was firstly transduced with miR-590-3p in a tet-on 3G induction system to become miR-H1 ESC line. Without doxycycline induction, miR-H1 can readily maintain their pluripotency and other typical characteristics of hESC (data not shown).

**Figure 2:**
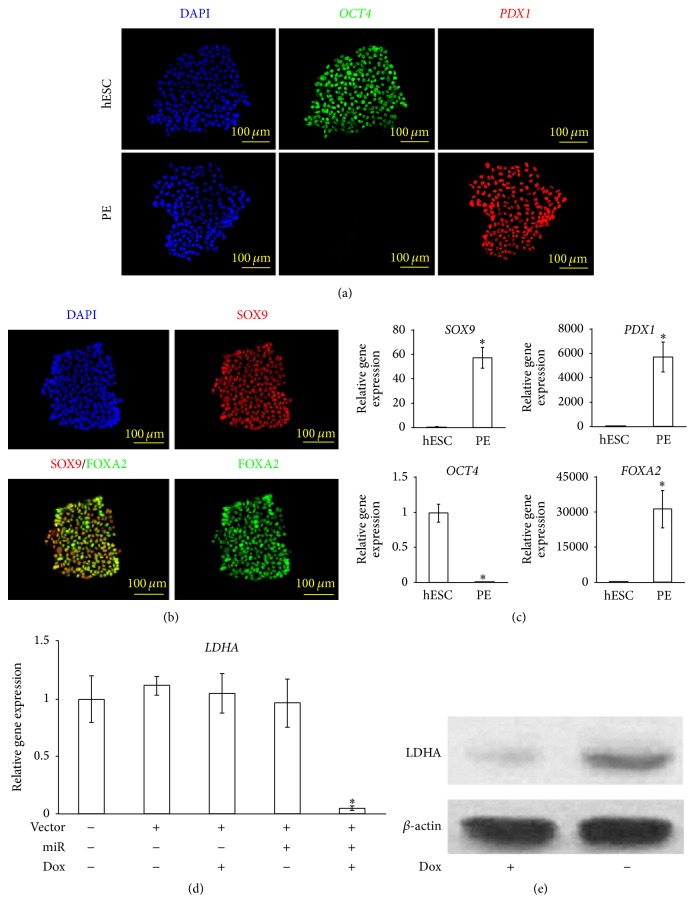
Characterization of hESC-derived PE and miR function. (a) miR-590-3p transfected H1 ESCs (miR-H1-ESC) were differentiated to pancreatic endoderm (PE) (see Section 2). Cells of undifferentiated miR-H1-ESC and differentiated PE (miR-PE) were subject to immunofluorescence analysis and stained for DAPI, OCT4, and PDX1. (b) miR-PE stained for DAPI, SOX9, and FOXA2. (c) Relative gene expression of* OCT4*,* PDX1*,* FOXA2*, and* SOX9* from miR-hESCs and miR-PE. All values are given as mean; error bar represents standard error. ^*∗*^ *p* < 0.05, compared with undifferentiated H1 hESC. (d) Real-time qPCR results of* LDHA* expression in H1-ESC with and without miR-590-3p transfection and doxycycline induction. All values are given as mean; error bar represents standard error. ^*∗*^ *p* < 0.05, compared with nontransduced H1 hESC. (e) Western blot analysis of LDHA in miR-PE cells with/without doxycycline induction.

Following the previously established four-stage differentiation protocol [15], we differentiated miR-H1 hESCs to pancreatic endoderm (miR-PE) (see Section 2). Using immunofluorescence, we were able to confirm the cells expressed appropriate markers throughout the differentiation. At the beginning of stage 1, H1 hESC expressed the pluripotency marker OCT4 [19] (Figure 2(a), top row). At stage 4, cells lost OCT4 and exhibited PDX1 expression (Figure 2(a), bottom row), a key pancreatic transcription factor [20]. We further confirmed the cell lineage by staining stage 4 hESC-derived cells with antibodies against FOXA2 and SOX9, another two signature transcription factors' characterization of pancreatic endoderm [20, 21], and they were both expressed in these cells (Figure 2(b)). Next, using quantitative RT-PCR, we further quantified the relative expression levels of the above marker genes, showing that the differentiated cells indeed had completely shut down* OCT4* expression and greatly increased mRNA levels of* PDX1*,* FOXA2*, and* SOX9* (Figure 2(c)). Therefore, the hESC-derived cells generated by our four-stage protocol closely matched committed pancreatic endoderm in the embryo [22].

Induced by doxycycline, miR-H1 ESCs showed significant decreasing of* LDHA* mRNA level compared to H1 ESCs without miR transduction. However, without doxycycline or miR-590-3p or both, no significant difference in* LDHA* mRNA level was found compared with the same control (Figure 2(d)). Using western blot, we also found a decreased LDHA protein level when miR-PE cells were induced by doxycycline (Figure 2(e)).

### 3.3. miR-PE Transplant Alleviated Diabetes Symptoms in Mice

**Figure 3:**
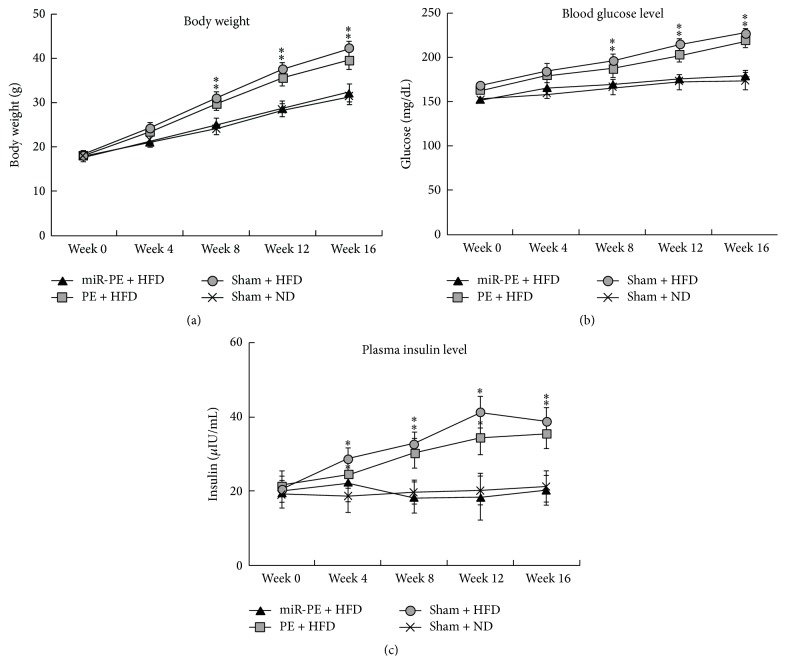
miR-PE transplant alleviated diabetes symptoms in mice. (a) Effect of HFD on body weight of mice. Body weight was measured 1 week after PE transplant for 16 weeks. (b) Blood glucose levels of mice. Blood glucose levels were monitored 1 week after PE transplant for 16 weeks. (c) Plasma insulin levels of mice. Plasma insulin concentrations were measured by ELISA 1 week after PE transplant for 16 weeks. All values are given as mean; error bar represents standard error. ^*∗*^ *p* < 0.05, compared with Sham + ND group.

hESC-derived PE (with or without miR-590-3p) were transplanted under the left kidney capsule of C57BL/6J mice (see Section 2); then their body weight, blood glucose, and plasma insulin levels were measured accordingly (week 0). We found that Sham + ND group showed a steady increase of body weight, while Sham + HFD group exhibited much faster weight gain. With PE transplant, animals in PE + HFD group could better control the weight gain by HFD, but animals with miR-PE transplant were almost insusceptible to HFD (Figure 3(a)). Blood glucose levels of all four experimental groups were almost the same at week 0, while mice in Sham + ND group maintained their blood glucose level, and animals in Sham + HFD group exhibit much faster increasing of blood glucose, presenting a typical T2D symptom of hyperglycemia. As expected, animals exposed to HFD but with PE transplant can slightly attenuate the increasing of blood glucose level, but the difference is not significant. However, mice that received miR-PE transplant showed much better tolerance of HFD and maintain their blood glucose level similar to those in Sham + ND group (Figure 3(b)). Plasma insulin levels in all four groups were also quite similar at week 0. However, Sham + HFD group started to produce more insulin as early as week 1. PE transplant in PE + HFD group somehow improved insulin management but the insulin level is still significantly higher than baseline Sham + ND group. On the other hand, the animals with miR-PE transplant could better cope with HFD and maintain plasma insulin at the baseline level (Figure 3(c)). The above results indicate transplant of miR-PE alleviating hyperglycemia and hyperinsulinemia induced by HFD.

### 3.4. miR-PE Transplant Maintains Normal *β*-Cells Mass and Size

**Figure 4:**
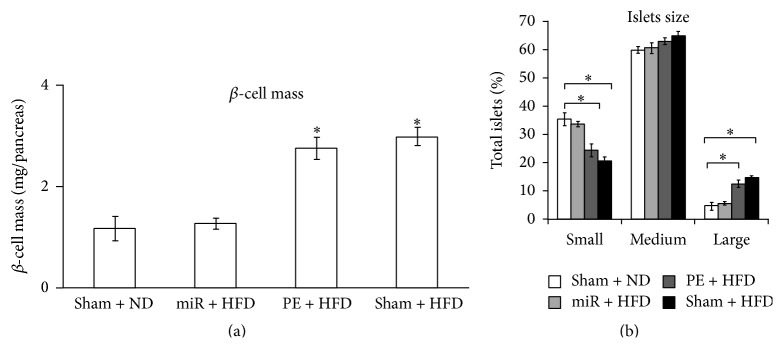
*β*-cell mass and islets size. (a) *β*-cell mass of animal at week 16. (b) Islets size of pancreas at week 16. All values are given as mean; error bar represents standard error. ^*∗*^ *p* < 0.05, compared with Sham + ND group.

It was well documented that the mass of *β*-cells adapts to the physiologic conditions such as glucose infusion, evident by markedly increased *β*-cells proliferation [23–25]. To investigate whether *β*-cells are affected by HFD, we further assessed the mass and size of *β*-cells. As shown in Figure 4(a), *β*-cell mass was dramatically increased (*p* < 0.05) in Sham + HFD and PE + HFD mice compared to Sham + ND mice, as a result of elevated insulin demands responding to glucose infusion caused by HFD. However, no significant elevation of *β*-cells mass was found in miR-PE + HFD mice. Moreover, the proportion of small islets in Sham + HFD and PE + HFD mice significantly decreased (*p* < 0.05), whereas large islets proportion significantly increased (*p* < 0.05) compared to Sham + ND groups (Figure 4(b)). The above results showed that miR-PE transplant can efficiently maintain the *β*-cells mass and islets size in normal range.

### 3.5. miR-PE Transplant Maintains Fat Content, Liver Weight and TG, and Plasma TG, FFA, and Leptin at a Baseline Level

Studies have shown underlying correlation between liver weight and obesity in T2D [26]. In order to understand how our treatment works on HFD, we also measured fat content, liver weight, and liver triglycerides (TGs) at the end of 16-week experiment. Sham + ND group was used as baseline control, and Sham + HFD group was used as T2D control.

**Figure 5:**
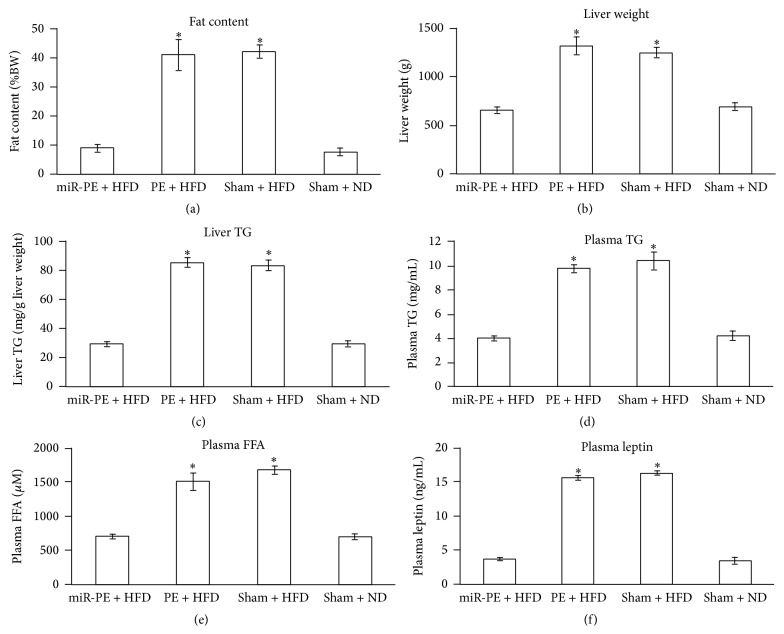
Fat content, liver weight and TG level, and plasma TG, FFA, and leptin level. (a) Fat content of animals at week 16. (b) Liver weight of animals at week 16. All values are given as mean; error bar represents standard error. ^*∗*^ *p* < 0.05, compared with Sham + ND group. (c) Liver TG level of animals at week 16. (d) Plasma TG level of animals at week 16. (e) Plasma FFA level of animals at week 16. (f) Plasma leptin level of animals at week 16. All values are given as mean; error bar represents standard error. ^*∗*^ *p* < 0.05, compared with Sham + ND group.

There is no significant difference in fat content between miR-PE + HFD and Sham + ND mice, while PE + HFD mice have significantly higher (*p* < 0.05) fat content than Sham + ND mice (Figure 5(a)). This suggested that mice with miR-PE transplant can better control fat deposition than PE + HFD mice. Mice with PE transplant only had slightly lower fat content than Sham + HFD mice, but the significance is weak (*p* < 0.01).

Liver is a major metabolic tissue for energy homeostasis such as synthesizing TG* de novo* from glucose or fatty acids obtained from diet and producing very low density lipoprotein (VLDL) to transport to the circulatory system and eventually to cells [27, 28]. In this context, liver weight and liver TG of mice that received miR-PE transplant were also maintained at similar level of Sham + ND mice. The measurement of Sham + HFD mice and PE + HFD mice, on the other hand, increased significantly compared to Sham + ND mice (Figures 5(b) and 5(c)).

In liver, FFAs are produced by the breakdown of TG obtained from either diet or cells like adipocytes as energy source of the animal body. With adequate plasma FFAs and TGs from diet, liver synthesizes VLDLs and transports them to adipocytes for storing fat or muscle cells for producing energy. Whereas during fasting plasma FFA and TG are insufficient to support energy consumption, liver then starts decomposing the stored fat. Elevated FFA influx is another factor causing TG accumulation in the liver, which is also related to T2D and obesity [29, 30]. We next measured plasma FFA and TG of the four groups of mice and found that both Sham + HFD and PE + HFD mice exhibited significantly increased levels of plasma FFA and TG (*p* < 0.05) after fasting, compared to Sham + ND mice. However, there is no significant difference of plasma TG and FFA between miR-PE + HFD and Sham + ND mice (Figures 5(d) and 5(e)).

Leptin is a hormone produced by adipose tissue and functions to stimulate glucose uptake and control energy expenditure by facilitating fatty acid oxidation and preventing lipid accumulation through activating AMP kinase [31] and therefore serves as another indicator of diabetes and obesity in our study. Plasma leptin concentration after fasting in both mice was significantly increased compared to Sham + ND mice (*p* < 0.05), while no significant difference was found in miR-PE + HFD mice (Figure 5(f)). Between Sham + HFD and PE + HFD mice, the latter group also exhibited lower plasma leptin level, but the significance is faint (*p* < 0.02).

### 3.6. miR-PE Attenuated Glucose Translocation and Enhanced Glycogenolysis and Gluconeogenesis

In order to further understand the effect of miR-PE transplant treatment at the molecular level, the mRNA and protein levels of several hormones and enzymes involved in gluconeogenesis and glycogenolysis in pancreas were evaluated. The expression level of these mRNA and protein in Sham + ND mice was used as baseline control.

**Figure 6:**
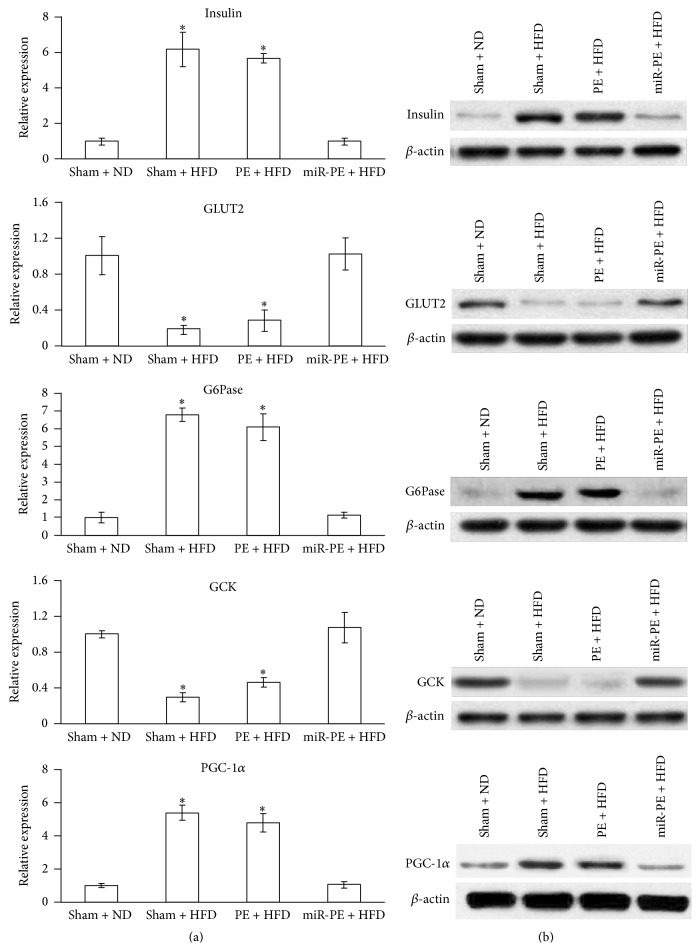
Assessment of hormones and enzymes involved in gluconeogenesis and glycogenolysis in pancreas. (a) mRNA levels of* insulin*,* GLUT2*,* G6Pase*,* GCK*, and* PGC-1α*. All values are given as mean; error bar represents standard error. ^*∗*^ *p* < 0.05, compared with Sham + ND group. (b) Western blot results of protein level of the same genes.

PGC-1*α* is a coactivator controlling gluconeogenesis by suppressing Akt activation, which is in turn repressed by insulin [32]. Insulin was found to block the actions of hormones involved in gluconeogenesis which also becomes resistant at diabetic state [33]. Significantly higher mRNA levels of insulin (*p* < 0.05) and PGC-1*α* (*p* < 0.05) were found in Sham + HFD and PE + HFD mice but not miR-PE + HFD mice, indicating that the insulin resistant status was better coped with in miR-PE + HFD mice. The level of G6Pase mRNA in Sham + HFD and PE + HFD mice was also markedly increased, while miR-PE + HFD mice remained at the baseline level, suggesting that glycogenolysis was significantly attenuated by miR-PE transplant in these mice. In particular, mRNA expressions of glucose transporter 2 (GLUT2) and glucokinase (GCK) were maintained at baseline level in miR-PE + HFD mice but downregulated in both Sham + HFD and PE + HFD mice. Since GCK expression responds to glucose intake and GCK functions to phosphorylate glucose to glucose-6-phosphate, the above result suggests reduced glucose translocation from blood to cells as well as glycolysis. To summarize, these results showed that miR-590-3p expressing PE transplant has excellent ability to maintain gluconeogenesis- and glycogenolysis-related hormones and enzymes at normal level. The protein levels of the above hormones and enzymes were also analyzed by western blot and were found to be consistent with their mRNA levels (Figure 6(b)).

## 4. Discussion

T2D patients were reported to exhibit progressively decreased number of pancreatic insulin-positive *β*-cells as well as their function [6]. Strategies to treat T2D usually lead to *β*-cells replacement, either from allogeneic donor or from derived stem cells.

Previous studies have demonstrated that functional *β*-cells can be derived from hESCs* in vitro*, and these cells showed positive results treating preexisting diabetes in mouse model [15, 16]. However, we showed that transplant of just hESC-derived PE exhibited insignificant effect preventing T2D development in long term HFD exposure. In the course of 16 weeks of HFD exposure, mice that received just PE transplant showed significant T2D symptoms of T2D compared to ND-control mice. In addition, when compared with T2D-positive control group (Sham + HFD) mice, no significant improvement was noticed. This possibly indicates that although *β*-cells derived from hESCs can attenuate preexisting diabetes symptoms to a certain extent by replenishing fresh and functional *β*-cells, these newly transplanted cells are still susceptible to loss of function and degeneration in long term HFD exposure and later T2D development.

In order to strengthen the effect of hESC-derived PE in T2D treatment, we aimed to strengthen transplant *β*-cells' survival and resistance to T2D environment. Our strategy is to maintain the normal gene regulation in *β*-cells. As mentioned, a group of “disallowed genes” were absent or at low expression level specifically in *β*-cells while being highly expressed in other mammalian tissues [9]. Among these genes, LHDA showed crucial importance as overexpression of LDHA in insulin secreting *β*-cells affects glucose-induced insulin secretion. Islets of individuals with diabetes also display an increase in the expression of LDHA [10–12]. Taken together, LDHA suppression is very crucial to *β*-cells function and survival in pancreatic tissue. To achieve this, we identified hsa-miR-590-3p which specifically targets LDHA mRNA in luciferase assay. We have also shown that hsa-miR-590-3p can readily suppress LDHA expression in HeLa cells, H1 ESCs, and H1 ESC derived PE cells. In order to prevent the possible negative effect of LDHA inhibition to hESC's pluripotency, we used a tet-on system to transfer the microRNA into hESC. Hsa-miR-590-3p was not activated by doxycycline until hESCs were successfully differentiated into desired PE cells and transplanted into animals.

In this study, we used a novel combinational treatment with stem cell-derived PE cells and LDHA-suppressing microRNA. Transplant of stem cell-derived PE not only filled up the loss of *β*-cells in T2D but also enhanced the insulin secretion. MicroRNA hsa-miR-590-3p, on the other hand, ensures LDHA was maintained at the normal low expression level in *β*-cells.

The miR-PE transplant showed significant attenuation of T2D symptoms in our mouse model. First of all, animals that received miR-PE transplant and HFD could well maintain their body weight and blood glucose and insulin level. There is no significant difference compared with Sham + ND mice. However, compared with PE + HFD mice, miR-PE + HFD mice showed significant improvement (Figure 3). In addition, miR-PE + HFD mice showed no sign of glycogenolysis and gluconeogenesis (Figure 6). These results well indicate that limiting LDHA expression in PE cells transplant by miR-590-3p efficiently improved the transplant's survival and function and eventually helped animals cope with long term HFD exposure and prevent T2D development.

In summary, our study has provided the first successful report of treating HFD induced T2D with conjunct stem cell and microRNA therapy in mouse model. Our treatment readily alleviated hyperglycemia and hyperinsulinemia and maintained body weight and other pancreatic functions at normal level. Therefore, our study has shown the importance of gene regulation in *β*-cells and strongly supported the feasibility for a similar therapy to treat T2D in human patients.

## References

[1] Barba C., Cavalli-Sforza T., Cutter J. (2004). Appropriate body-mass index for Asian populations and its implications for policy and intervention strategies. *The Lancet*.

[2] Barness L. A., Opitz J. M., Gilbert-Barness E. (2007). Obesity: genetic, molecular, and environmental aspects. *American Journal of Medical Genetics A*.

[3] Surwit R. S., Kuhn C. M., Cochrane C., McCubbin J. A., Feinglos M. N. (1988). Diet-induced type II diabetes in C57BL/6J mice. *Diabetes*.

[4] Ikemoto S., Takahashi M., Tsunoda N., Maruyama K., Itakura H., Ezaki O. (1996). High-fat diet-induced hyperglycemia and obesity in mice: differential effects of dietary oils. *Metabolism: Clinical and Experimental*.

[5] Astrup A., Buemann B., Western P., Toubro S., Raben A., Christensen N. J. (1994). Obesity as an adaptation to a high-fat diet: evidence from a cross-sectional study. *The American Journal of Clinical Nutrition*.

[6] Ng S.-F., Lin R. C. Y., Laybutt D. R., Barres R., Owens J. A., Morris M. J. (2010). Chronic high-fat diet in fathers programs *β*-cell dysfunction in female rat offspring. *Nature*.

[7] Schuit F. C., In 't Veld P. A., Pipeleers D. G. (1988). Glucose stimulates proinsulin biosynthesis by a dose-dependent recruitment of pancreatic beta cells. *Proceedings of the National Academy of Sciences of the United States of America*.

[8] Abderrahmani A., Plaisance V., Lovis P., Regazzi R. (2006). Mechanisms controlling the expression of the components of the exocytotic apparatus under physiological and pathological conditions. *Biochemical Society Transactions*.

[9] Pullen T. J., Rutter G. A. (2013). When less is more: the forbidden fruits of gene repression in the adult *β*-cell. *Diabetes, Obesity and Metabolism*.

[10] Hellman B., Idahl L. A., Sehlin J., Taljedal I. B. (1975). Influence of anoxia on glucose metabolism in pancreatic islets: lack of correlation between fructose 1,6 diphosphate and apparent glycolytic flux. *Diabetologia*.

[11] Sekine N., Cirulli V., Regazzi R. (1994). Low lactate dehydrogenase and high mitochondrial glycerol phosphate dehydrogenase in pancreatic *β*-cells: potential role in nutrient sensing. *Journal of Biological Chemistry*.

[12] Ainscow E. K., Zhao C., Rutter G. A. (2000). Acute overexpression of lactate dehydrogenase-A perturbs *β*-cell mitochondrial metasbolism and insulin secretion. *Diabetes*.

[13] Francese R., Fiorina P. (2010). Immunological and regenerative properties of cord blood stem cells. *Clinical Immunology*.

[14] Fiorina P., Voltarelli J., Zavazava N. (2011). Immunological applications of stem cells in type 1 diabetes. *Endocrine Reviews*.

[15] Kroon E., Martinson L. A., Kadoya K. (2008). Pancreatic endoderm derived from human embryonic stem cells generates glucose-responsive insulin-secreting cells in vivo. *Nature Biotechnology*.

[16] Rezania A., Bruin J. E., Riedel M. J. (2012). Maturation of human embryonic stem cell-derived pancreatic progenitors into functional islets capable of treating pre-existing diabetes in mice. *Diabetes*.

[17] Pagliuca F. W., Millman J. R., Gürtler M. (2014). Generation of functional human pancreatic *β* cells in vitro. *Cell*.

[18] Ludwig T. E., Bergendahl V., Levenstein M. E., Yu J., Probasco M. D., Thomson J. A. (2006). Feeder-independent culture of human embryonic stem cells. *Nature Methods*.

[19] Niwa H., Miyazaki J.-I., Smith A. G. (2000). Quantitative expression of Oct-3/4 defines differentiation, dedifferentiation or self-renewal of ES cells. *Nature Genetics*.

[20] Jensen J. (2004). Gene regulatory factors in pancreatic development. *Developmental Dynamics*.

[21] Jørgensen M. C., Ahnfelt-Rønne J., Hald J., Madsen O. D., Serup P., Hecksher-Sørensen J. (2007). An illustrated review of early pancreas development in the mouse. *Endocrine Reviews*.

[22] Zorn A. M., Wells J. M. (2007). Molecular basis of vertebrate endoderm development. *International Review of Cytology*.

[23] Finegood D. T., Scaglia L., Bonner-Weir S. (1995). Dynamics of *β*-cell mass in the growing rat pancreas: estimation with a simple mathematical model. *Diabetes*.

[24] Efrat S. (1996). Genetic engineering of *β*-cells for cell therapy of diabetes: cell growth, function, and Immunogenicity. *Diabetes Reviews*.

[25] Porat S., Weinberg-Corem N., Tornovsky-Babaey S. (2011). Control of pancreatic *β* cell regeneration by glucose metabolism. *Cell Metabolism*.

[26] Yang S. Q., Lin H. Z., Lane M. D., Clemens M., Diehl A. M. (1997). Obesity increases sensitivity to endotoxin liver injury: implications for the pathogenesis of steatohepatitis. *Proceedings of the National Academy of Sciences of the United States of America*.

[27] Strable M. S., Ntambi J. M. (2010). Genetic control of de novo lipogenesis: role in diet-induced obesity. *Critical Reviews in Biochemistry and Molecular Biology*.

[28] McDevitt R. M., Bott S. J., Harding M., Coward W. A., Bluck L. J., Prentice A. M. (2001). De novo lipogenesis during controlled overfeeding with sucrose or glucose in lean and obese women. *American Journal of Clinical Nutrition*.

[29] Adams L. A., Angulo P., Lindor K. D. (2005). Nonalcoholic fatty liver disease. *Canadian Medical Association Journal*.

[30] Golay A., Swislocki A. L. M., Chen Y.-D. I., Reaven G. M. (1987). Relationships between plasma-free fatty acid concentration, endogenous glucose production, and fasting hyperglycemia in normal and non-insulin-dependent diabetic individuals. *Metabolism*.

[31] Gil-Campos M., Cañete R., Gil A. (2004). Hormones regulating lipid metabolism and plasma lipids in childhood obesity. *International Journal of Obesity*.

[32] Puigserver P., Rhee J., Donovan J. (2003). Insulin-regulated hepatic gluconeogenesis through FOXO1-PGC-1*α* interaction. *Nature*.

[33] Puigserver P., Spiegelman B. M. (2003). Peroxisome proliferator-activated receptor-*γ* coactivator 1*α* (PGC-1*α*): transcriptional coactivator and metabolic regulator. *Endocrine Reviews*.

